# (*E*)-1-(4-Bromo­phen­yl)-3-(2-fur­yl)prop-2-en-1-one

**DOI:** 10.1107/S1600536809014299

**Published:** 2009-04-22

**Authors:** Jin-Ming Gao, Jian-Chun Qin, Ya-Mei Zhang, Guang-Jun Li

**Affiliations:** a College of Science, Northwest A&F University, Yangling, Shaanxi 712100, People’s Republic of China; b Center for Experiments & Education Technology, Linyi Normal University, Linyi, Shandong 276005, People’s Republic of China

## Abstract

In the title compound, C_13_H_9_BrO_2_, the benzene and furan rings form a dihedral angle of 44.35 (14)°. The crystal packing exhibits no significantly short inter­molecular contacts.

## Related literature

For the crystal structure of a related compound, see: Li *et al.* (1992 ▶). For general background, see: Yadav & Mashram (2001 ▶).
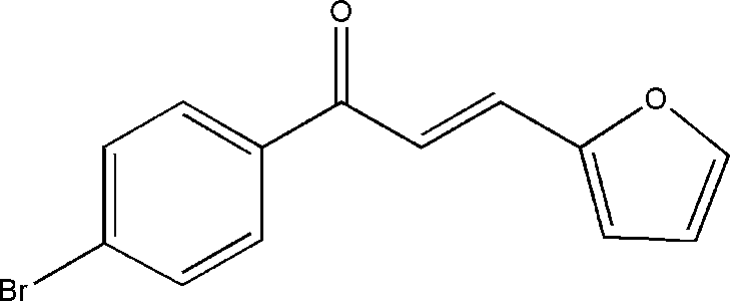


## Experimental

### 

#### Crystal data

C_13_H_9_BrO_2_
*M* *_r_* = 277.11
Monoclinic, 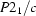

*a* = 14.172 (4) Å
*b* = 14.064 (4) Å
*c* = 5.8002 (18) Å
β = 98.353 (4)°
*V* = 1143.8 (6) Å^3^
*Z* = 4
Mo *K*α radiation
μ = 3.57 mm^−1^
*T* = 298 K
0.48 × 0.40 × 0.34 mm

#### Data collection

Bruker SMART APEX CCD area-detector diffractometer
Absorption correction: multi-scan (*SADABS*; Sheldrick, 1996 ▶) *T*_min_ = 0.279, *T*_max_ = 0.376 (expected range = 0.220–0.297)
5665 measured reflections
2015 independent reflections
1344 reflections with *I* > 2σ(*I*)
*R*_int_ = 0.028

#### Refinement

*R*[*F* ^2^ > 2σ(*F* ^2^)] = 0.046
*wR*(*F* ^2^) = 0.133
*S* = 1.07
2015 reflections
145 parameters
H-atom parameters constrained
Δρ_max_ = 0.35 e Å^−3^
Δρ_min_ = −0.45 e Å^−3^

### 

Data collection: *SMART* (Siemens, 1996 ▶); cell refinement: *SAINT* (Siemens, 1996 ▶); data reduction: *SAINT*; program(s) used to solve structure: *SHELXS97* (Sheldrick, 2008 ▶); program(s) used to refine structure: *SHELXL97* (Sheldrick, 2008 ▶); molecular graphics: *SHELXTL* (Sheldrick, 2008 ▶); software used to prepare material for publication: *SHELXTL*.

## Supplementary Material

Crystal structure: contains datablocks I, global. DOI: 10.1107/S1600536809014299/cv2540sup1.cif

Structure factors: contains datablocks I. DOI: 10.1107/S1600536809014299/cv2540Isup2.hkl

Additional supplementary materials:  crystallographic information; 3D view; checkCIF report

## Figures and Tables

**Fig. 1.**
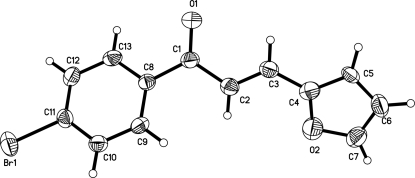
The molecular structure of (I) showing the atomic numbering scheme and 30% probability displacement ellipsoids.

## supplementary crystallographic information

### Comment

Reactions under solvent-free or so-called dry media conditions are especially appealing as they provide an opportunity to work with open vessels, thus avoiding the risk of high pressure development and with the possibility of upscaling the reactions to larger scale (Yadav & Mashram, 2001).

In continuation of our ongoing program directed to the development of environmentally benign methods of chemical synthesis, we describe in this paper a user-friendly, solvent-free protocol for the synthesis of chalcones starting from the fragrant aldehydes and fragrant ketones in the presence of NaOH under solvent-free conditions. Using this method, which can be considered as a a general method for the synthesis of chalcones, we obtained the title compound, (I). We present here its crystal structure.

In (I) (Fig. 1), the bond lengths and angles are normal and comparable to those observed in the reported compound (Li *et al.*, 1992). The benzene and furan rings form a dihedral angle of 44.35 (14)°. The crystal packing exhibits no significantly short intermolecular contacts.

### Experimental

Furan-2-carbaldehyde (0.5 mmol) and 4-bromoacetophenone (0.5 mmol), NaOH (0.5 mmol) were mixed in 50 ml flash under sovlent-free condtions. After stirring for 5 min at 293 K, the resulting mixture was washed with water for several times for removing NaOH, and recrystallized from ethanol, and afforded the title compound as a crystalline solid. Elemental analysis: calculated for C_13_H_9_BrO_2_: C 56.34, H 3.27%; found: C 56.38, H 3.35%.

### Refinement

All H atoms were placed in geometrically idealized positions (C—H 0.93 Å) and treated as riding on their parent atoms, with *U*_iso_(H) = 1.2 *U*_eq_(C).

### Crystal data

**Table:**

C_13_H_9_BrO_2_	*F*(000) = 552
*M**_r_* = 277.11	*D*_x_ = 1.609 Mg m^−^^3^
Monoclinic, *P*2_1_/*c*	Mo *K*α radiation, λ = 0.71073 Å
*a* = 14.172 (4) Å	Cell parameters from 1645 reflections
*b* = 14.064 (4) Å	θ = 2.9–24.0°
*c* = 5.8002 (18) Å	µ = 3.57 mm^−^^1^
β = 98.353 (4)°	*T* = 298 K
*V* = 1143.8 (6) Å^3^	Block, yellow
*Z* = 4	0.48 × 0.40 × 0.34 mm

### Data collection

**Table:**

Bruker SMART APEX CCD area-detector diffractometer	2015 independent reflections
Radiation source: fine-focus sealed tube	1344 reflections with *I* > 2σ(*I*)
graphite	*R*_int_ = 0.028
φ and ω scans	θ_max_ = 25.0°, θ_min_ = 2.1°
Absorption correction: multi-scan (*SADABS*; Sheldrick, 1996)	*h* = −16→16
*T*_min_ = 0.279, *T*_max_ = 0.376	*k* = −16→16
5665 measured reflections	*l* = −4→6

### Refinement

**Table:**

Refinement on *F*^2^	Primary atom site location: structure-invariant direct methods
Least-squares matrix: full	Secondary atom site location: difference Fourier map
*R*[*F*^2^ > 2σ(*F*^2^)] = 0.046	Hydrogen site location: inferred from neighbouring sites
*wR*(*F*^2^) = 0.133	H-atom parameters constrained
*S* = 1.07	*w* = 1/[σ^2^(*F*_o_^2^) + (0.0633*P*)^2^ + 0.7234*P*] where *P* = (*F*_o_^2^ + 2*F*_c_^2^)/3
2015 reflections	(Δ/σ)_max_ < 0.001
145 parameters	Δρ_max_ = 0.35 e Å^−^^3^
0 restraints	Δρ_min_ = −0.45 e Å^−^^3^

### Special details

**Table:**

Geometry. All e.s.d.'s (except the e.s.d. in the dihedral angle between two l.s. planes) are estimated using the full covariance matrix. The cell e.s.d.'s are taken into account individually in the estimation of e.s.d.'s in distances, angles and torsion angles; correlations between e.s.d.'s in cell parameters are only used when they are defined by crystal symmetry. An approximate (isotropic) treatment of cell e.s.d.'s is used for estimating e.s.d.'s involving l.s. planes.
Refinement. Refinement of *F*^2^ against ALL reflections. The weighted *R*-factor *wR* and goodness of fit *S* are based on *F*^2^, conventional *R*-factors *R* are based on *F*, with *F* set to zero for negative *F*^2^. The threshold expression of *F*^2^ > σ(*F*^2^) is used only for calculating *R*-factors(gt) *etc*. and is not relevant to the choice of reflections for refinement. *R*-factors based on *F*^2^ are statistically about twice as large as those based on *F*, and *R*- factors based on ALL data will be even larger.

### Fractional atomic coordinates and isotropic or equivalent isotropic displacement parameters (Å^2^)

**Table:**

	*x*	*y*	*z*	*U*_iso_*/*U*_eq_	
Br1	−0.41996 (3)	0.37809 (5)	0.12851 (11)	0.0898 (3)	
O1	0.0212 (2)	0.3772 (2)	−0.1992 (5)	0.0770 (10)	
O2	0.2477 (3)	0.3452 (3)	0.5655 (7)	0.0839 (10)	
C1	0.0029 (3)	0.3749 (3)	−0.0007 (7)	0.0524 (10)	
C2	0.0790 (3)	0.3714 (3)	0.2017 (8)	0.0542 (11)	
H2	0.0637	0.3577	0.3487	0.065*	
C3	0.1694 (3)	0.3878 (3)	0.1760 (8)	0.0558 (11)	
H3	0.1811	0.4054	0.0282	0.067*	
C4	0.2501 (4)	0.3809 (3)	0.3540 (8)	0.0609 (12)	
C5	0.3401 (3)	0.4042 (3)	0.3343 (8)	0.0566 (12)	
H5	0.3612	0.4298	0.2032	0.068*	
C6	0.3966 (3)	0.3830 (4)	0.5455 (10)	0.0765 (15)	
H6	0.4620	0.3928	0.5810	0.092*	
C7	0.3415 (4)	0.3471 (4)	0.6837 (9)	0.0754 (14)	
H7	0.3609	0.3263	0.8353	0.091*	
C8	−0.0983 (3)	0.3758 (3)	0.0412 (7)	0.0450 (9)	
C9	−0.1271 (3)	0.4102 (3)	0.2437 (7)	0.0505 (10)	
H9	−0.0817	0.4314	0.3646	0.061*	
C10	−0.2221 (3)	0.4132 (3)	0.2679 (8)	0.0550 (11)	
H10	−0.2411	0.4373	0.4032	0.066*	
C11	−0.2888 (3)	0.3802 (3)	0.0900 (8)	0.0524 (10)	
C12	−0.2635 (3)	0.3453 (3)	−0.1120 (8)	0.0581 (11)	
H12	−0.3096	0.3227	−0.2298	0.070*	
C13	−0.1670 (3)	0.3443 (3)	−0.1383 (7)	0.0527 (10)	
H13	−0.1487	0.3225	−0.2765	0.063*	

### Atomic displacement parameters (Å^2^)

**Table:**

	*U*^11^	*U*^22^	*U*^33^	*U*^12^	*U*^13^	*U*^23^
Br1	0.0493 (3)	0.1156 (6)	0.1045 (5)	0.0100 (3)	0.0115 (3)	−0.0091 (4)
O1	0.065 (2)	0.120 (3)	0.0475 (19)	0.000 (2)	0.0141 (16)	0.0032 (18)
O2	0.075 (2)	0.091 (3)	0.085 (3)	−0.0059 (19)	0.005 (2)	0.001 (2)
C1	0.058 (2)	0.057 (3)	0.043 (2)	0.002 (2)	0.0087 (19)	0.002 (2)
C2	0.057 (3)	0.056 (3)	0.052 (2)	0.0015 (19)	0.013 (2)	0.004 (2)
C3	0.057 (3)	0.063 (3)	0.049 (2)	0.010 (2)	0.014 (2)	0.004 (2)
C4	0.069 (3)	0.061 (3)	0.052 (3)	0.016 (2)	0.006 (2)	0.000 (2)
C5	0.043 (2)	0.081 (3)	0.049 (2)	0.006 (2)	0.019 (2)	0.012 (2)
C6	0.046 (3)	0.091 (4)	0.093 (4)	−0.003 (3)	0.015 (3)	−0.008 (3)
C7	0.075 (3)	0.086 (4)	0.062 (3)	0.006 (3)	−0.005 (3)	0.001 (3)
C8	0.049 (2)	0.044 (2)	0.043 (2)	0.0012 (18)	0.0058 (18)	0.0046 (18)
C9	0.056 (3)	0.055 (2)	0.039 (2)	−0.0066 (19)	0.0008 (19)	−0.0005 (18)
C10	0.060 (3)	0.059 (3)	0.046 (2)	0.008 (2)	0.009 (2)	−0.003 (2)
C11	0.046 (2)	0.052 (2)	0.059 (3)	0.0096 (19)	0.006 (2)	0.000 (2)
C12	0.054 (3)	0.061 (3)	0.056 (3)	0.004 (2)	−0.005 (2)	−0.004 (2)
C13	0.067 (3)	0.051 (2)	0.040 (2)	0.008 (2)	0.007 (2)	−0.0048 (19)

### Geometric parameters (Å, °)

**Table:**

Br1—C11	1.905 (4)	C6—C7	1.300 (7)
O1—C1	1.217 (5)	C6—H6	0.9300
O2—C4	1.331 (6)	C7—H7	0.9300
O2—C7	1.405 (6)	C8—C9	1.385 (6)
C1—C2	1.475 (6)	C8—C13	1.391 (6)
C1—C8	1.489 (6)	C9—C10	1.374 (6)
C2—C3	1.331 (6)	C9—H9	0.9300
C2—H2	0.9300	C10—C11	1.374 (6)
C3—C4	1.428 (7)	C10—H10	0.9300
C3—H3	0.9300	C11—C12	1.365 (6)
C4—C5	1.337 (6)	C12—C13	1.398 (6)
C5—C6	1.395 (7)	C12—H12	0.9300
C5—H5	0.9300	C13—H13	0.9300
			
C4—O2—C7	107.1 (4)	C6—C7—H7	125.7
O1—C1—C2	121.4 (4)	O2—C7—H7	125.7
O1—C1—C8	119.8 (4)	C9—C8—C13	119.0 (4)
C2—C1—C8	118.7 (4)	C9—C8—C1	123.4 (4)
C3—C2—C1	120.6 (4)	C13—C8—C1	117.5 (4)
C3—C2—H2	119.7	C10—C9—C8	120.7 (4)
C1—C2—H2	119.7	C10—C9—H9	119.6
C2—C3—C4	126.2 (4)	C8—C9—H9	119.6
C2—C3—H3	116.9	C9—C10—C11	119.3 (4)
C4—C3—H3	116.9	C9—C10—H10	120.3
O2—C4—C5	108.9 (4)	C11—C10—H10	120.4
O2—C4—C3	124.5 (4)	C12—C11—C10	121.9 (4)
C5—C4—C3	126.5 (4)	C12—C11—Br1	118.5 (3)
C4—C5—C6	107.9 (4)	C10—C11—Br1	119.5 (3)
C4—C5—H5	126.1	C11—C12—C13	118.7 (4)
C6—C5—H5	126.1	C11—C12—H12	120.7
C7—C6—C5	107.7 (4)	C13—C12—H12	120.7
C7—C6—H6	126.2	C8—C13—C12	120.3 (4)
C5—C6—H6	126.2	C8—C13—H13	119.8
C6—C7—O2	108.5 (5)	C12—C13—H13	119.8

## References

[bb1] Li, Z.-D., Huang, L.-Z., Su, G.-B. & Wang, H.-Y. (1992). *Chin. J. Struct. Chem.* **11**, 1–4.

[bb2] Sheldrick, G. M. (1996). *SADABS* University of Göttingen, Germany.

[bb3] Sheldrick, G. M. (2008). *Acta Cryst.* A**64**, 112–122. 10.1107/S0108767307043930 18156677

[bb4] Siemens (1996). *SMART* and *SAINT* Siemens Analytical X-ray Instruments Inc., Madison, Wisconsin, USA.

[bb5] Yadav, J. S. & Mashram, H. M. (2001). *Pure. Appl. Chem.* **73**, 199–203.

